# Individuals with a history of Cesarean or Induced abortion combined with allergies will significantly increase their risk of endometriosis: A prospective case-control study

**DOI:** 10.12669/pjms.42.6.13847

**Published:** 2026-06

**Authors:** Shanshan Mei, Jie Ding, Zhexin Ni, Chaoqin Yu

**Affiliations:** 1 Shanshan Mei, MD. Department of Obstetrics and Gynecology, The First Affiliated Hospital of Zhejiang Chinese Medical University, (Zhejiang Provincial Hospital of Chinese Medicine), Zhejiang, China; 2 Jie Ding, MD. Department of Gynecology Department of Traditional Chinese Medicine, The First Affiliated Hospital of Naval Military Medical University, Shanghai, China; 3 Zhexin Ni, PHD. Academy of Military Medical Sciences, Beijing, China; 4 Chaoqin Yu, MD. Department of Gynecology Department of Traditional Chinese Medicine, The First Affiliated Hospital of Naval Military Medical University, Shanghai, China

**Keywords:** Allergy History, Case control study, Cesarean section, Endometriosis, Epidemiology, Induced abortion

## Abstract

**Objective:**

To investigate the association between delivery/abortion methods, allergy history, and endometriosis (EM) risk.

**Methodology:**

This prospective case-control study was conducted at the First Affiliated Hospital of Naval Medical University (Shanghai Changhai Hospital), China, from 2018 to 2024. We enrolled 1498 EM patients and 452 non-EM controls. Associations between clinical variables (delivery modes, abortion, allergy history) and EM incidence were analyzed using multivariate logistic regression to adjust for confounders.

**Results:**

Cesarean section increased EM risk approximately 2-fold (OR 2.043, 95% CI: 1.489-2.804); this risk remained significant after excluding scar EM (OR 1.944, 95% CI: 1.414-2.674). Induced abortion also correlated with a 2-fold increased risk of EM (OR 2.262, 95% CI: 1.529-3.347). Furthermore, allergic patients with a history of induced abortion had a significantly higher likelihood of developing deep infiltrating endometriosis (DIE).

**Conclusion:**

Cesarean section and induced abortion independently increase EM risk. Moreover, induced abortion combined with an allergy history elevates the risk of developing DIE, highlighting the synergistic effect of surgical trauma and immune status on EM pathogenesis.

## INTRODUCTION

About 10-15% of women in the world who are of reproductive age suffer with endometriosis (EM), a widespread chronic inflammatory disease that is dependent on estrogen.^1^ Approximately ninety percent of patients experience some degree of pelvic pain, including dyspareunia, non-menstrual pelvic pain, and dysmenorrhea, among other clinical symptoms. Furthermore, about 26% of patients have infertility, which has a significant negative influence on their reproductive health and quality of life.^2^ The most severe kind, Deep Infiltrating Endometriosis (DIE), significantly reduces natural reproductive potential by often altering pelvic architecture, causing severe dyspareunia, and causing persistent pelvic inflammation.^3^

Laparoscopy and postoperative pathology confirmation are still required for the gold standard of EM diagnosis, despite the severe risk to women’s health. Due to the lack of accurate, non-invasive diagnostic biomarkers, EM diagnosis is sometimes much later than expected. China may see delays of up to 13 years, compared to the global average of 7-10 years.^4^ The necessity and significance of determining high-risk variables for EM are highlighted by this diagnostic dilemma. This could provide innovative methods for disease prevention in addition to facilitating early detection and intervention among high-risk populations.

The pathophysiology of endometriosis is still not well understood. In 1927, Sampson introduced the retrograde menstruation idea, which is still the most frequently recognized view today. Although endometriosis is only 10-15% common, this explanation is unable to explain why over 90% of people experience retrograde menstruation. This suggests that in ectopic sites, the effective implantation, proliferation, and penetration of retrograde endometrial tissue must be facilitated by extra crucial elements. Environmental exposures, endogenous estrogen levels, and genetic susceptibility have all been shown to be important risk factors for EM in previous studies.^5^-^7^

Current research has primarily evaluated the isolated impacts of immunological factors such as baseline immune status and inflammatory mediators or surgical interventions on the pathogenesis of EM. However, the mechanisms governing their synergistic interactions and combined contributions to disease susceptibility remain largely unexplored. For example, although it has been shown that cesarean sections directly cause abdominal incisional endometriosis,^8^ there is still disagreement among academics over whether or not they are a risk factor for pelvic endometriosis on their own. Furthermore, critical clinical inquiries remain unaddressed, particularly regarding whether a history of induced abortion influences the incidence and pathological phenotyping of EM, and whether a patient’s immunological profile exacerbates this risk following such procedures. Notably, allergy disorders are more common in EM patients, according to some epidemiological research and clinical observations,^9^ suggesting that immunological dysregulation may be a major factor in the pathophysiology of EM. Still missing, though, is rigorous research data about the inherent relationships and interactions between induced abortion, cesarean section, and allergy history and EM risk.

With this in mind, the current study was performed to methodically examine the relationship between the risk of endometriosis and a history of induced abortion, cesarean section, and allergies. The relationship between these variables and particular pathological subtypes of endometriosis was also examined. The results are anticipated to offer new views on clarifying the intricate, multifaceted pathophysiology of endometriosis as well as evidence-based medical insights for risk assessment, early detection, and therapeutic decision-making.

## METHODOLOGY

This was a single-center prospective case-control study. The study was performed at a Grade A tertiary hospital in China: The First Affiliated Hospital of Naval Medical University (Shanghai Changhai Hospital). A total of 1,742 female patients with pathologically proven endometriosis and 461 patients diagnosed with ovarian cysts unrelated to endometriosis or primary dysmenorrhea unrelated to endometriosis, who visited the center between 2018 and 2024, were included. The subsequent patients were omitted from the study: a. Patients with a verified diagnosis of adenomyosis, b. Patients with significant concurrent cardiac, pulmonary, hepatic, renal, hematological, or neurological conditions, c. Patients who are pregnant or lactating. Before administering the questionnaire, all survey personnel received standardized training; clinical data collection was conducted by gynecologists with more than three years of clinical experience who had completed the training. All patient data were electronically gathered and analyzed by designated individuals upon the completion of data collection.

Based on medical consultation records, we excluded 14 patients in the observation group for lacking histopathological diagnosis, 92 patients with a diagnosis of adenomyosis, and 138 patients without a clear pathological typology. In the control group, 9 patients who were diagnosed with adenomyosis were excluded from the study. Eventually, 1498 patients were enrolled in the observational group and 452 patients in the control group.

### Questionnaire survey:

In this study, two questionnaires were developed. The sociological demographic characteristics questionnaire included: age, education level, labor level, exercise status, history of alcohol consumption, smoking history, sexual life, marriage status, allergy status, and other systemic diseases. The reproductive characteristics questionnaire included: number of conceptions, number of births, number of cesarean surgeries, number of normal deliveries, number of induced abortions, number of medication abortions, and EM pathological type.

### Statistical analyses:

Student t-tests and chi-square tests were applied to analyze differences in sociological demographic characteristics and reproductive characteristics as appropriate. Logistic regression was used to estimate the ratio (OR) with 95% confidence interval for the association between risk factors and EM. SPSS 26.0 software was used for statistical processing and analysis. The results were presented as mean (standard deviation) and value (percentage). p-value less than 0.05 was considered statistically significant.

### Ethical approval:

This study passed the ethical review of the Medical Ethics Committee of Shanghai Changhai Hospital (Ethical code: CHEC2018-195; dated December 30, 2018). All participants agree with this study. All subjects in this study participated voluntarily and signed the informed consent form.

## RESULTS

The baseline characteristics were comparable between observational and control groups. No significant difference was identified in age, alcohol and tobacco consumption, physical work status and sexual history between two groups. However, our data suggested that a greater proportion of patients in observational group had higher education and married compared to the control group. Furthermore, the prevalence of frequent exercise, systemic disease and allergy history was increased in the observational group compared to the control group (Table-I).

**Table-I:** Comparison of sociodemographic characteristics in case and control groups.

Social demographic characteristic	Case (n=1498)	Control(n=452)	P vaule
Age(Y)	36.46(6.871)	34.99(7.257)	0.193
***Education***			
Illiterate/Primary	88(5.9)	24(5.3)	0.005
Guidance/High school	370(24.7)	132(29.2)	
Junior college	326(21.8)	121(26.8)	
Regular college course	714(47.7)	175(38.7)	
Exercise habits			＜0.001
Yes	569(38)	113(25)	
No	929(62)	339(75)	
***Occupational physical activity***			
Sedentary work	563(37.6)	159(35.2)	0.08
Light physical activity	743(49.6)	225(49.8)	
Moderate physical activity	146(9.7)	60(13.3)	
Heavy physical labor	46(3.1)	8(1.8)	
Smoking history			0.208
Yes	18(1.2)	9(2)	
No	1480(98.8)	443(98)	
Drinking history			0.376
Yes	95(6.3)	34(7.5)	
No	1403(93.7)	418(92.5)	
Having a history of allergies			0.004
Yes	178(11.9)	32(7.1)	
No	1320(88.1)	420(92.9)	
Having a history of other systemic diseases			＜0.001
Yes	263(17.6)	23(5.1)	
No	1235(82.4)	429(94.9)	
Having a history of sexual intercourse			0.653
Yes	1353(90.3)	405(89.6)	
No	145(9.7)	47(10.4)	
Has a history of marriage			＜0.001
Yes	1213(79.1)	285(68.3)	
No	320(20.9)	132(31.7)	

As for the reproductive characteristics, we examined the effect of different delivery methods on the risk of EM in the married population only, in order to avoid the potential confounding factor of marital status. No statistical difference in number of conceptions and births was identified between two groups. However, increased portion of patients received cesarean surgery in the observational group compared to the control group (Table-II). Next we measured the abortion frequency between two groups and discovered that increased proportion of patients received surgical abortion in the observational group compared to the control group while there was no statistical difference in terms of medication abortion identified between two groups (Table-III).

**Table-II:** Comparison of way of giving birth in case and control groups(married).

	Case (n=1213)	Control(n=320)	P vaule
Number of gravidarum	2.26(2.493)	2.47(2.913)	0.248
Number of births	0.79(0.73)	0.83(0.95)	0.505
Number of cesarean section	0.32(0.535)	0.23(0.677)	0.022
Number of eutocia	0.47(0.689)	0.6(0.745)	0.004
Having a history of cesarean section			＜0.001
Yes	457(37.7)	153(47.8)	
No	756(62.3)	167(52.2)	
Having a history of eutocia			＜0.001
Yes	352(29.0)	56(17.5)	
No	861(71.0)	264(82.5)	

**Table-III:** Comparison of way of abortion in case and control groups.

	Case (n=1498)	Control(n=452)	P vaule
Number of induced abortion	0.46(0.915)	0.37(0.912)	0.045
Number of medical abortion	0.08(0.363)	0.12(0.436)	0.098
Having a history of induced abortion			＜0.001
Yes	422(28.2)	92(20.4)	
No	1076(71.8)	360(79.6)	
Having a history of medical abortion			0.136
Yes	95(6.3)	38(8.4)	
No	1403(93.7)	414(91.6)	

In multivariate adjusted analysis, we observed that cesarean surgery increased the risk of EM by almost 2-fold (OR 2.043, 95% CI: 1.489-2.804) which was remained close to 2-fold (OR 1.944, 95% CI: 1.414 -2.674) after removing the association with scar endometriosis. Similarly, abortion was also associated with a 2-fold increase risk of EM(OR 2.262, 95% CI: 1.529-3.347) (Table-IV).

**Table-IV:** Comparison of way of giving birth and way of abortion in case and control groups based on bivariate and multivariate logistic regression.

Variable	Unadjusted		Adjusted^a^	
	P value	OR (CI 95%)	P value	OR (CI 95%)
Having a history of cesarean section	＜0.001	2.196	＜0.001	2.043
		1.620 to 2.977		1.489 to 2.804
Having a history of cesarean section (No scar endometriosis)	＜0.001	2.100	＜0.001	1.944
		1.548 to 2.894		1.414 to 2.674
Having a history of induced abortion	＜0.001	1.535	＜0.001	2.262
		1.189 to 1.981		1.529 to 3.347

a:Adjusted for frequency matching variables of reference age and gravidarum number as well as birth number, abortions number, physical exercise, education degree and allergy.

In an exploratory analysis to identify the association between different EM risk factors and different pathological phenotypes, our results revealed no significant difference among different EM subtypes in patients with or without cesarean surgery history. Whereas more DIE occurred in patients with a history of abortion compared to patients without abortion history (Table-V). Finally, our analysis suggested that patients with allergy were more likely to develop EM, especially if they had abortion history (Table-VI).

**Table-V:** Association between cesarean section and induced abortion and endometriosis typing.

	Having a history of induced abortion		P vaule
Types of endometriosis	Yes(n=422)	No(n=1076)	＜0.001
Peritoneal endometriosis	44(10.4)	127(11.8)	
Ovarian Endometriosis	289(68.5)	817(75.9)	
Deep Infiltrating Endometriosis	49(11.6)	57(5.3)	
Other Endometriosis	40(9.5)	75(7.0)	
	*Having a history of cesarean section*		*P vaule*
Types of endometriosis	Yes(n=355)	No(n=1143)	0.059
Peritoneal endometriosis	45(12.7)	126(11.0)	
Ovarian Endometriosis	250(70.4)	856(74.9)	
Deep Infiltrating Endometriosis	22(6.2)	84(7.3)	
Other Endometriosis	38(10.7)	77(6.7)	

**Table-VI:** Association between allergy and endometriosis.

	Case (n=422)	Control(n=92)	P vaule^a^
Having a history of allergy			0.013
Yes	40(9.4)	1(1.0)	
No	382(90.6)	91(99.0)	
	*Case (n=355)*	*Control(n=56)*	*P vaule^b^*
Having a history of allergy			0.207
Yes	43(12.1)	3(5.3)	
No	312(87.9)	53(94.7)	
	*Case (n=178)*	*Case (n=32)*	*P vaule^C^*
Having a history of induced abortion			0.021
Yes	40(22.5)	1(3.1)	
No	138(77.5)	31(96.9)	
Having a history of cesarean section			0.103
Yes	43(27.5)	3(9.4)	
No	135(72.5)	29(90.6)	

a:People with a history of induced abortion; b:People with a history of cesarean section; C:People with a history of allergy; Tip: Check with continuous corrected chi-square.

## DISCUSSION

This study identified cesarean section and induced abortion as risk factors for endometriosis (EM), while medical abortion demonstrated no significant association with the development of EM. Additionally, using a large-sample prospective case-control design, this study first identifies the synergistic clinical risk of developing deep infiltrating endometriosis (DIE) by combining surgical interventions (induced abortion) with a patient’s baseline immune dysregulation (allergy history).

A mechanism of EM pathogenesis involves the implantation of endometrial tissue outside the uterine cavity. Cesarean section and induced abortion both result in damage to the endometrium in the uterine cavity, which subsequently elevates the risk of ectopic implantation of endometrial tissue.^10^ Further research is needed to explore the underlying biological mechanisms that link these surgical interventions to the increased risk of EM. Understanding these pathways could inform clinical practices and guide preventative strategies for at-risk individuals.

Cesarean section is an invasive surgical procedure performed on the lower uterine segment. Endometrial fragments that are actively shed during surgery may be retained at the incision site and subsequently invade surrounding tissues. Estrogen plays a role in the development of EM associated with scars on the abdominal wall.^11^ Suction catheters are frequently used to remove decidual tissue and gestational sacs during artificial abortions. Small curettes are commonly employed to meticulously scrape the uterine horns and cavity walls, thereby preventing residual embryonic tissue. These procedures result in damage to the endometrium, thereby increasing the probability of endometrial fragments being dispersed into the abdominal cavity.^12^

Typically, the microenvironments of the abdominal and pelvic cavities maintain a very steady condition. This pelvic microenvironmental balance may be altered by exogenous stressors. The risk of pelvic EM is unavoidably increased after cesarean section surgeries due to the use of suction instruments and surgical techniques that easily spread endometrial fragments into the pelvic environment via the uterine incision.^13^ Moreover, the healing process following cesarean section surgery requires the participation of multiple cell adhesion molecules, cytokines, and chemokines in wound repair. These factors accumulate extensively at the site of trauma, altering the overall microenvironment of the pelvic cavity. They serve as the core coordinators governing the interaction between endometrial fragments and other pelvic tissues. This imbalance in the microenvironment may promote the onset and progression of EM.^14^ Studies indicate that activated platelets after cesarean section contribute to tissue regeneration by enhancing ERβ receptor expression in endometriotic stromal cells and stimulating COX-2 synthesis in monocytes, endothelial cells, and stromal cells.^15^ Increased levels of intercellular adhesion molecule-1 (ICAM-1) hinder natural killer cell function, hence expediting the onset of EM.^15^ Moreover, research indicates that immune cells, including macrophages and T lymphocytes, are involved in the tissue repair process after both cesarean sections and induced abortions.^16^

Macrophages facilitate local homeostasis restoration by conveying signals of hypoxia, cellular demise, and iron accumulation at the injury site. Preclinical evidence supports that surgical trauma alters the local pelvic microenvironment by driving macrophage polarization toward the M2 phenotype and disrupting the regulatory T-cell balance, processes that actively facilitate the ectopic proliferation of endometrial cells.^17^ Consequently, the inflammatory cascade and immune evasion initiated by cesarean sections and induced abortions entail various high-risk factors that exacerbate the progression of EM, which is consistent with the heightened clinical incidence observed in our patient cohort.

Our findings demonstrate that cesarean section correlates with a heightened incidence of pelvic EM, aligning with prior research,^18^ although no link to specific pathological subtypes was found. This study identified a link between induced abortion and deep infiltrating endometriosis (DIE). Induced abortion produces significant micro-fragments of endometrial tissue via vacuum aspiration, enabling their infiltration into the pelvic and abdominal cavities. These pieces may circulate in peritoneal fluid and disperse to locations such as the uterosacral ligaments, rectovaginal septum, vaginal fornix, rectum, colon, bladder, and ureters.^19^

Furthermore, negative pressure inhalation may increase the probability of endometrial fragments spreading through lymphatic and vascular routes. Recent research suggests that, in contrast to apoptotic endometrial cells found in menstrual blood, endometrial tissue fragments expelled following stressful operations may preserve greater biological activity. When these highly active cells are introduced by surgical instruments or intrauterine pressure gradients into deep pelvic structures-such as the rectouterine pouch or uterosacral ligaments, which are common sites for deep endometriosis-they acquire the potential to implant ectopically, endure, and commence invasive growth.^20^ Additionally, the creation of a postoperative inflammatory environment and the avoidance of immune surveillance are significant factors that facilitate the progression of DIE. Any intrauterine surgery essentially represents a sterile trauma, eliciting a localized acute inflammatory response. The secretion of major pro-inflammatory cytokines (including TNF-α, IL-1β, and IL-6) and chemokines markedly modifies the pelvic microenvironment.^21^

This ‘pro-inflammatory state’ increases the attachment efficiency of ectopic endometrial cells in the pelvis by upregulating the expression of cell adhesion molecules, such as ICAM-1. Simultaneously, it promotes the release of angiogenic elements such as vascular endothelial growth factor (VEGF), thus ensuring the necessary blood supply for the sustenance and proliferation of ectopic lesions.^22^ Moreover, chronic inflammation may lead to localized immunological dysregulation, hindering the identification and elimination of ectopic endometrial cells by macrophages and natural killer (NK) cells. This immune evasion is a prevalent mechanism for the development of all types of EM. The severe inflammatory cascade initiated by induced abortion may accelerate the evolution of EM.^23^

This study further indicates that persons with an allergic constitution exhibit a heightened susceptibility to EM, particularly among those with a history of induced abortion. This is the first epidemiological study to clinically link an allergic constitution with a heightened susceptibility to DIE following an induced abortion. This phenomenon may arise from the heightened activity of the immune system in individuals with allergy histories, making them more susceptible to surgical triggers and hence increasing vulnerability to EM. An imbalance between TH1 and TH2 responses in allergic individuals results in markedly increased IgE levels and precipitates an allergic condition, suggesting that the Th1-Th2 equilibrium in allergic populations is more precarious than in healthy individuals.^24^

Post-abortion stress disturbs the TH1-TH2 equilibrium, resulting in increased TH2 activity. Th1 and Th2 cytokines directly govern angiogenesis, indirectly stimulate the release of additional cytokines in the microenvironment by affecting cell growth and differentiation, and engage in the angiogenic process by modulating the expression of specific receptors related to endothelial cell proliferation and migration.^25^ Research demonstrates that Th2-type cytokines are markedly increased in individuals with EM, and the Th1/Th2 ratio is associated with illness progression.^26^ The trauma of miscarriage may exacerbate the naturally delicate TH1-TH2 equilibrium in allergic individuals, fostering conditions favorable to the initiation and advancement of EM.

### Limitations:

Firstly, as a single-center, hospital-based study, inherent selection bias exists, and the enrolled subjects may not fully represent the general population, which limits the external validity of our findings. Secondly, unidentified potential confounding variables may be present; thirdly, the presence of multiple pathological subtypes may introduce data bias; furthermore, the sample size of patients with a history of allergies was relatively limited; and our current data failed to differentiate between specific allergy subtypes including asthma and allergic rhinitis. Thus, to validate these findings and eliminate potential demographic biases, future large-scale, multi-center prospective cohort studies are essential.

## CONCLUSIONS

Our study suggested that cesarean section and abortion were independent risk factors for developing EM and abortion was associated with the development of DIE. Finally, we found that patients with allergy history were at higher risk for developing EM after Induced abortion, suggesting that the development of EM was influenced by both the procedure and the patient’s immune status. The completion of this study not only provided valuable reference to clinic but also shed light upon understanding the pathogenesis of EM.

### Recommendations:

Future studies should specifically focus on addressing this gap by investigating the distinct impacts of particular allergy phenotypes on EM susceptibility; additionally, we did not perform a stratified analysis of the relationship between secondary cesarean section and EM.

### Role of Funding Source:

This study was supported by the Zhejiang Provincial Natural Science Foundation [Project No.: ZCLQN25H2702]; the National Natural Science Foundation of China [Project No.: 82304962]; the Zhejiang University of Chinese Medicine Institutional Research Project [Project No.: 2024FSYYZQ04]; and the Collaborative Project on Major and Complex Diseases Integrating Chinese and Western Medicine: ‘Endometriosis’ [Project No.: ZDYN-2024-A-0158]. The content is solely the responsibility of the authors and does not necessarily represent the official view of the Naval Military Medical University. The funding sources were not involved in the conduct of research and/or preparation of the manuscript.

### Authors’ Contribution:

**SM:** Conducted the data analysis and drafted the manuscript, with **JD** making significant contributions to the analysis.

**SM and JD:** Are both considered as co-first authors.

**ZN:** Assisted with the analysis, provided constructive discussion and was responsible for the accuracy of this study.

**CY:** Contributed to the conception of the study and was responsible for the integrity of this study.
